# Cabergoline Failure and a Spontaneous Pregnancy in a Microprolactinoma with High Prolactin Levels

**DOI:** 10.3390/jpm12122061

**Published:** 2022-12-14

**Authors:** Andrei Adrian Tica, Daniela Dumitrescu, Irina Tica, Corina Neamţu, Vlad Iustin Tica, Cristiana Iulia Dumitrescu, Oana Sorina Tica

**Affiliations:** 1 Department of Pharamacology, University of Medicine and Pharmacy, 200349 Craiova, Romania; 2 Department of Radiology, University of Medicine and Pharmacy, 200349 Craiova, Romania; 3 IIIrd Medical Department, Faculty of Medicine, University “Ovidius” of Constanţa, 900470 Constanţa, Romania; 4 Columna Medical Center, 021522 Bucharest, Romania; 5 Donna Medical Center, 021522 Bucharest, Romania; 6 Department of Obstetrics and Gynecology, Faculty of Medicine, University “Ovidius” of Constanţa, 900470 Constanţa, Romania; 7 Department “Mother and Child”, University of Medicine and Pharmacy, 200349 Craiova, Romania

**Keywords:** prolactinoma, prolactin, dopamine agonist therapy (DA), cabergoline, pregnancy, GnRH, LH, FSH, estradiol

## Abstract

We report a particular case of a spontaneously occurring pregnancy in a long-term amenorrheic patient due to a prolactinoma with high serum prolactin (PRL) following the failure of dopamine agonist therapy (DA) for infertility. Initially, clinical, laboratory, and genital ultrasounds were normal, but the serum PRL was 10,074 μIU/mL (n.v.: 127–637 μIU/mL), the PEG fraction was 71% (laboratory cut-off > 60%), and luteinizing hormone (LH) was significantly lower. An MRI revealed a pituitary tumor of 12.8/10 mm with a subacute intratumoral hemorrhage. DA was initiated, and menstrual bleeding reappeared with a reduction in the tumor’s volume to 1.9/2.2 mm at 12 months. Two years later, the patient renounced DA and follow-ups. After another 2 years, she became spontaneously pregnant. Serum PRL was 18,325 μIU/mL, and an MRI revealed a microprolactinoma of 2.1/2 mm. The patient gave birth to a normal baby at term, and she breastfed for six months, after which she asked for ablactation, and DA was administered. This case highlights the possibility of the occurrence of a normal pregnancy during a long period of amenorrhea induced by a microprolactinoma with a high level of serum PRL, even if DA fails to correct infertility. There was no compulsory relationship between the tumoral volume’s evolution and the evolution of its lactophore activity. The hypogonadotrophic hypogonadism induced by high PRL was mainly manifested by low LH, and in this situation, normal levels of FSH and estradiol do not always induce follicle recruitment and development without abnormalities in the ovary ultrasound.

## 1. Introduction

Despite the relatively small number of prolactin (PRL)-secreting cells in the anterior pituitary gland (15–25%) [1], prolactinomas are the most frequent local tumors [2,3], consisting of over 40% of hypophyseal adenomas [4]. Primarily occurring as microadenomas (<1 cm) [5], their incidence is estimated to be around 100 per 1 million of the population [4]; they are also more frequently diagnosed in women in the 20–40-year-old age range [6].

Hyperprolactinemia is responsible for menstrual irregularities and even amenorrhea, but prolactinomas—even those with very high values of plasma levels of hormones—are not a serious problem in restoring fertility, with dopamine agonist therapy (DA) being very effective [2,7,8].

However, the present case proves that without DA or with DA failure, completely unexpected gestation can occur in amenorrheic women after a long period due to a microprolactinoma with high PRL plasma levels, and if no other abnormal conditions exist, with adequate monitoring, the pregnancy outcome can be very good.

## 2. Case Report

A 31-year-old patient who had been married for 1.5 years presented for infertility treatment. She had amenorrhea for 7 years without galactorrhea. The patient’s written consent was obtained to publish her case.

Her medical history revealed menarche at 16 years old followed by spaniomenorrhea and amenorrhea. She remembered the presence of elevated levels of serum prolactin and menstrual response to bromocriptine, but she also recognized inconsistent treatment with bromocriptine for as long as 8 years after menarche. She could not provide any medical records.

The clinical and gynecological ultrasound examinations revealed no abnormalities.

The total plasma prolactin level was 10,074 μIU/mL (normal laboratory values: 127–637 μIU/mL) with an extracted fraction after polyethylene glycol (PEG) precipitation of 7153 μIU/mL (71%; laboratory cut-off > 60%). The other blood hormonal tests in the follicular phase showed the following: estradiol: 26.2 pg/mL (12.4–341.5 pg/mL); follicle-stimulating hormone (FSH): 4 mIU/mL (3.5–7.7 mIU/mL); luteinizing hormone (LH): 0.6 mIU/mL (2.4–11.4 mIU/mL); 17-OH progesterone: 0.4 ng/mL (0.1–0.8 ng/mL); dehydroepiandrosterone sulfate (DHEA-S): 267 mg/dL (98.8–340 mg/dL); insulin-like growth factor 1 (IGF 1): 113 ng/mL (109–271 ng/mL), thyroid-stimulating hormone (TSH): 2.55 mIU/mL (0.27–4.2 mIU/mL); and cortisol: 236 nmol/L (172–497 nmol/L).

**Figure 1:**
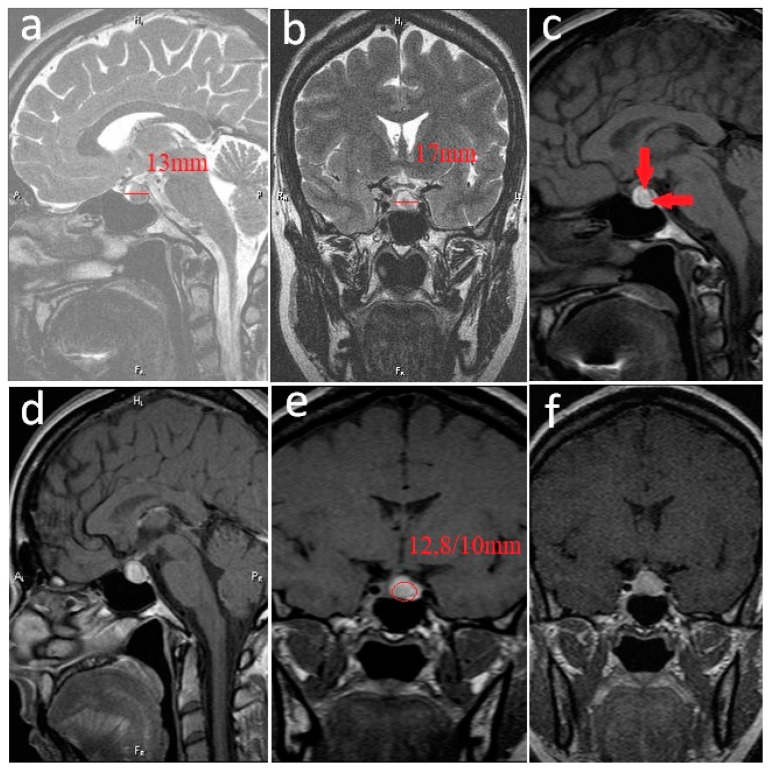
**Prolactinoma with bleeding areas inside. Pituitary MRI at diagnosis.** (**a**). Noncontrast sagittal T2-weighted image. (**b**). Noncontrast coronal T2-weighted image. (**c**). Noncontrast sagittal T1-weighted image—diffuse bleeding (hyper T1—arrows). (**d**). Postcontrast sagittal T1-weighted image. (**e**). Postcontrast coronal T1-weighted image. (**f**). Postcontrast coronal T1-weighted image. Pituitary fossa—anteroposterior diameter: 13 mm; transverse diameter: 17 mm; oval-shaped lesion 12.8/10 mm; heterogeneous high-signal T2-weighted (**a**,**b**); T1-weighted (**c**,**d**), located in the adenohypophysis with high signal in T1; weighted areas corresponding to the subacute intratumoral hemorrhage due to the presence of methemoglobin, heterogeneous enhancement (**e**,**f**), extending superiorly to the pituitary fossa (**b**,**d**,**e**,**f**) with mild compression of the optic chiasma (**b**,**d**,**f**).

Hypothalamic–pituitary magnetic resonance imaging (MRI) revealed an oval-shaped adenohypophyseal lesion measuring 12.8/10 mm with a high signal in T1-weighted areas corresponding to a subacute intratumoral hemorrhage. The tumor extended superiorly to the pituitary fossa, with mild compression of the optic chiasma (Figure 1).

The patient did not complain of any symptoms except amenorrhea, and her ophthalmologic assessment proved to be within normal limits despite the MRI aspect.

All other investigations of the patient and her partner failed to reveal other causes of infertility.

The endocrinologist decided to administer cabergoline, starting with 0.25 mg twice weekly and progressively increasing the dose. Menstrual bleeding reappeared at 1 mg twice weekly but with irregular periods. Serum PRL decreased to 110 µIU/mL at 6 months and was maintained at 12 months. After 12 months of treatment, the MRI showed a pituitary tumor with an important reduction in volume compared with the initial measurement—φ = 1.9/2.2 mm.

DA was stopped after around two years, and amenorrhea reoccurred. Despite the medical advice and disappointment of not having achieved a pregnancy, the patient stopped the PRL survey, and more importantly, she did not follow up on the tumor evolution by imaging.

Two years after DA interruption and reoccurrence of amenorrhea (again without galactorrhea), she returned with a positive pregnancy test, with ultrasonography showing a six-week gestational sac with an embryo presenting cardiac activity. 

Affirmatively, over the previous two years, the patient renounced any specific medication for infertility and asked for no more medical exams, resignedly accepting not being able to conceive. She did not mention any other significant health conditions or special medication after the DA period, either.

The plasma prolactin level was 18,325 μIU/mL (normal laboratory values for the first trimester of pregnancy are 189–4051 μIU/mL) with an extracted fraction after PEG precipitation of 12,650 μIU/mL (69%; laboratory cut-off > 60%).

**Figure 2:**
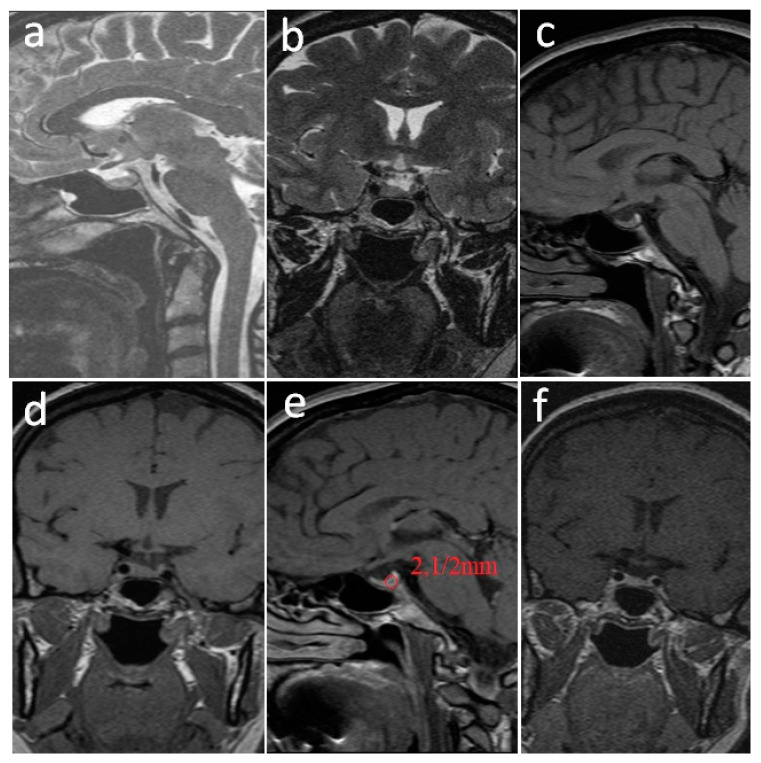
**Pituitary MRI performed 4 years later when the patient was 6 weeks pregnant.** (**a**). Non-contrast sagittal T2-weighted image. (**b**). Non-contrast coronal T2-weighted image. (**c**). Non-contrast sagittal T1-weighted image. (**d**). Non-contrast coronal T1-weighted image. (**e**). Post-contrast sagittal T1-weighted image. (**f**). Post-contrast coronal T1-weighted image. Favorable evolution of the pituitary adenoma located in the left parapituitary in moderate high-signal T2-weighted (**a**,**b**), low-signal T1-weighted (**c**,**d**), and non-gadolinium enhanced (**e**,**f**) with dimensions reduced to 2.1/2 mm.

The MRI again revealed a microprolactinoma of 2.1/2 mm (Figure 2).

Together with the endocrinology department, the decision was made to simply survey the pregnancy without DA.

Regular ophthalmological examinations revealed no modifications, and the patient did not complain of any neurological symptoms, such as headaches, nausea, or diplopia, during the entirety of the pregnancy.

At 39 weeks, she gave birth to a boy weighing 3620 g, Apgar 9, who showed no abnormalities. The PRL was 19,365 μIU/mL (normal laboratory values: 127–637 μIU/mL) and an MRI performed 2 days after delivery showed no modifications during pregnancy.

We decided, together with the endocrinologist, to allow breastfeeding; therefore, there was again no DA therapy.

The patient asked for ablactation 6 months later, and 1 mg weekly (0.5 mg twice a week) of cabergoline was administered. PRL at that moment was 18,978 μIU/mL (normal laboratory values: 127–637 μIU/mL). Under DA, lactation rapidly stopped, and the first menstrual bleeding appeared 4 weeks later, but again, the next ones occurred at irregular intervals. 

We decided, together with the endocrinologist, to allow breastfeeding; therefore, there was again no DA therapy.

The patient asked for ablactation 6 months later, and 1 mg weekly (0.5 mg twice a week) of cabergoline was administered. Under DA, lactation rapidly stopped, and the first menstrual bleeding appeared 4 weeks later, but again, the next ones occurred at irregular intervals. 

**Figure 3:**
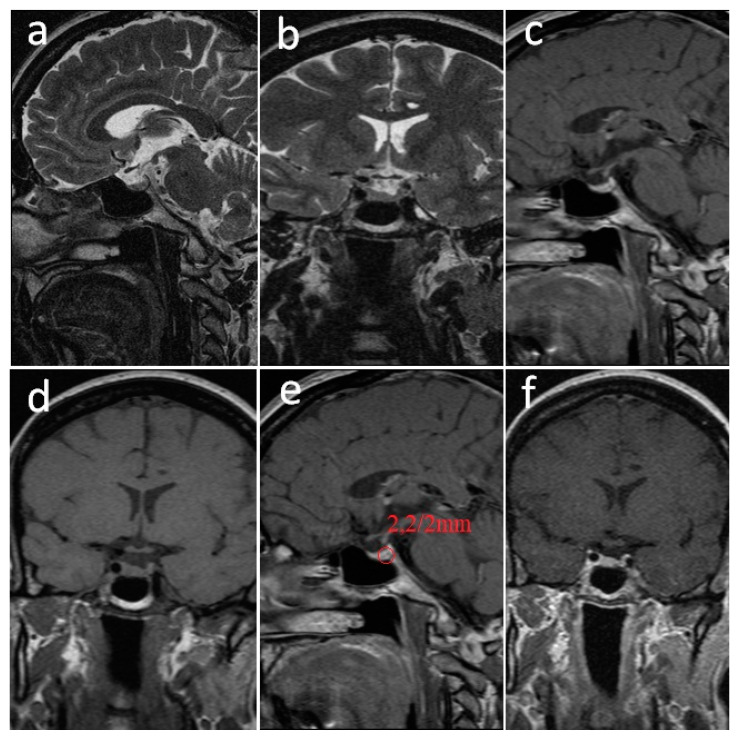
**Pituitary MRI performed 6 months after delivery.** (**a**). Non-contrast sagittal T2-weighted image. (**b**). Non-contrast coronal T2-weighted image. (**c**). Non-contrast sagittal T-weighted image. (**d**). Non-contrast coronal T1-weighted image. (**e**). Post-contrast sagittal T1-weighted image. (**f**). Post-contrast coronal T1-weighted image. The imaging aspect was the same as that at the beginning of the pregnancy; the pituitary adenoma was located in the left parapituitary in moderate high-signal T2-weighted (**a**,**b**), low-signal T1-weighted (**c**,**d**), and non-gadolinium enhanced (**e**,**f**). The diameter of the pituitary adenoma was 2/2 mm.

At the first menstruation, the serum PRL value was 475 μIU/mL (normal laboratory values: 127–637 μIU/mL); LH: 2.3 mIU/mL (2.4–11.4 mIU/mL). The MRI revealed a pituitary microadenoma with similar dimensions to that at the beginning of the pregnancy: 2/2 mm (Figure 3).

## 3. Discussion

In non-pregnant patients, it is generally accepted that a PRL blood concentration higher than 3000 μIU/mL corresponds with the existence of a prolactinoma, although there are several drugs used in psychiatry that can induce such concentrations (phenothiazines, risperidone, antidepressants, etc.) [6,9]. Concentrations over 5000 μIU/mL are characteristic of macroprolactinomas (>1 cm) [6,10]. However, in our case, the PRL serum being higher than 10,000 μIU/mL was the result of a microprolactinoma; its dimensions of over 1 cm at the first MRI evaluation were secondary to the intratumoral hemorrhage. Even if baseline serum PRL concentrations are strongly correlated with tumor dimension [11], high prolactin plasma levels are not compulsory or secondary to a big tumor (>1 cm), rather they are due to increased lactotroph activity, as other published cases have proven [12]. 

Furthermore, the tumor we described had similar dimensions after 12 months of DA and at the moment of pregnancy registration: 1.9/2.2 mm vs. 2.1/2 mm (and it is very probable that it had a similar volume with the “healthy” and “active” part of the pituitary tumor, without its hemorrhagic content, at the first MRI evaluation). However, serum PRL almost doubled at pregnancy registration compared with its value at the first evaluation of the patient, proving that prolactinoma can significantly increase its hormone release without significantly modifying its volume. As previously mentioned, this is a result of increased lactophore activity. 

It is true that the highest value (18,325 μIU/mL) was measured at 6 weeks of pregnancy, but it is generally accepted that PRL significantly rises after 6 weeks of pregnancy [13,14], reaching 756–4473 μIU/mL at the end of the first trimester [14,15]. Therefore, the gestation had no or very little impact on maternal serum PRL.

Of all three prolactin isoforms—monomeric: “little-prolactin” (23 kDa), dimeric: “big-prolactin” (40–60 kDa), and macroprolactin: “big-big-prolactin” (150 kDa)—only the former is active, able to bind PRL receptors (PRLRs) and generate specific physiological effects [16]. In this study, the active monomeric PRL fraction recovered after precipitation with polyethylene glycol (PEG) was 71% (the laboratory cut off for “absent” macroprolactinemia > 60%), meaning it was “true” hyperprolactinemia. It is important to differentiate “true” from “false” hyperprolactinemia, which can “escape” from clinical diagnosis because in the second condition, patients present minimal or no menstrual irregularities [17]. In these cases, the major fraction of plasmatic PRL consists of limited or completely “inactive” macroprolactin [17].

Hyperprolactinemia induces hypogonadotropic hypogonadism in both sexes [18,19]; in women, it causes oligomenorrhea or amenorrhea, anovulation, galactorrhea, and infertility [6,20].

Despite the very high PRL levels, amenorrhea in our patient was not associated with galactorrhea. This occurs in more than half of hyperprolactinemic patients because the hypogonadism induced by high PRL deprives the breast of adequate estrogenic and progesteronic support for milk secretion [15]. This is also the case for prolactinomas during or just before menopause [15].

During her first visit, our patient had blood concentrations of estradiol and FSH in the lower part of the normal ranges for the follicular phase of the menstrual cycle: 26.2 pg/mL (12.4–233.1 pg/mL) and 4 mIU/mL (3.5–12.5 mIU/mL), respectively. It is important to mention that “normal” FSH and estradiol did not induce follicle recruitment and development, so the ovary ultrasound was normal.

The hypogonadotropic hypogonadism was only truly proven by the significantly reduced blood concentrations of LH: 0.6 mIU/mL (2.4–11.4 mIU/mL). These very low plasma levels of LH were the result of the inhibition of (pulsatile) secretion of the gonadotropin-releasing hormone (GnRH) induced by high plasma PRL [21], a fact proven by LH returning to normal under DA. Caraty et al. [21] proved that the pulsatile secretion of GnRH blockade completely inhibits pulsatile LH secretion, while the FSH concentration fell more slowly and maintained a constitutive secretion [21,22]. Furthermore, the frequency of GnRH release is responsible for the selective secretion of LH and FSH: a rapid pulse increases the α and β LH-genes, and a slow pulse increases FSH-β gene transcription [23].

Despite the very well-known inhibitory effect of hyperprolactinemia on GnRH release, the intimate mechanism remains unclear [24]. Although a direct effect of PRL on GnRH neurons is possible [25], this should have a secondary physiological significance since only a small percentage of these neurons have PRL receptors (PRLRs) [13,26].

The missing link seems to be kisspeptins, which other studies have also considered [26,27]. Kisspeptins act as neuromediators and bind specific receptors—KISS1Rs, also known as G protein-coupled receptor 54 (GPR54), which are located on GnRH neurons—thereby increasing their activity [13,14]. Most kiss neurons express PRLRs [13,28]. After binding these receptors, PRL suppresses Kiss1 gene expression, thereby inhibiting kisspeptin synthesis and their release [13]. In the hypothalamus, kiss neurons are primarily located in the infundibular nucleus (arcuate nucleus in non-human species) and secondarily in the preoptic area (POA) [29]. Three-quarters of kiss neurons from the infundibular nucleus co-express neurokinin B and dynorphin, which constitute kisspeptin–neurokinin–dynorphin (KNDy) neurons [28,29,30,31]. Neurokinin B and dynorphin are absent in POA kiss neurons [31]. 

KNDy neurons, together with glial cells (and very likely other intercalary neurons) [32], form a complex network fundamentally involved in the generation of pulsatile GnRH secretion [24]. This theory is sustained by the presence of a great number of axo-somatic, axo-dendritic and axo-axonic contacts of KNDy neurons with GnRH neurons [32]. 

Neurokinin B binds specific receptors, such as neurokinin B receptors, situated on kiss neurons, thereby increasing kisspeptin release, or on GnRH-neurons, thereby stimulating GnRH secretion [24,31].

By contrast, dynorphin, after binding kappa opioid receptors, decreases the release of kisspeptin and/or GnRH from the respective neurons [24,31].

KNDy neurons seem to consist not only of the modulatory interface between prolactin and GnRH, but they are also fundamentally involved in the negative feedback induced by peripheral sex steroids on GnRH secretion because, unlike GnRH-neurons, which do not, KNDy neurons express receptors for estrogen (ERα), progesterone (PR), and testosterone (androgen receptors: AR) [31,33].

Furthermore, KNDy neurons act as headquarters for fertility modulation depending on stress, nutrition, or drugs because, again, unlike GnRH-neurons, they express glucocorticoid receptors [34,35], leptin receptors [36], and, as mentioned above, kappa opioid receptors [30,36].

Pituitary prolactinomas usually do not constitute serious problems concerning treatment for infertility [2]; both bromocriptine and cabergoline (DA) are permitted to achieve a large number of pregnancies and newborns [37,38]. Furthermore, Liu et al. [39] also reported an autophagic and apoptotic effect on lactotroph cells with a direct effect on prolactinoma disappearance.

Regarding the duration of DA therapy in women with prolactinomas who want to become pregnant, there are no strict recommendations. We considered 2 years of DA without pregnancy to be therapy failure. Because more than half of pregnancies occur in the first 6 months of treatment, Crosignani et al. [40] consider therapy failure to be one year of DA therapy for infertility (induced by high PRL levels) without pregnancy. However, there was a recently published case of prolactinoma in which DA therapy was maintained for three years without success in achieving a pregnancy [41]. 

The medical literature reveals that DA therapy should be continued in patients with prolactinomas for at least two years, and it can be discontinued when normalization of serum PRL levels and the disappearance of pituitary tumors are achieved [1,18,42]. Supplementarily, if PRL levels are kept suppressed by a small dose of DA (<0.5 mg/week), then DA can be arrested [42]. After cabergoline withdrawal, the reoccurrence of high levels of serum PRL in microprolactinomas is as high as 50% [42], a fact that imposes a continuation of follow-up for at least another 24 months [4]. 

If DA fails to restore fertility, clomiphene or aromatase inhibitors (AIs) can be added, especially when DA does not sufficiently decrease the PRL levels to allow ovulation [43], but in the present case, cabergoline induced a decrease in PRL levels of up to 110 μIU/mL after 6 months of treatment.

However, our case demonstrates that in amenorrheic women with high prolactin plasma levels secondary to a microprolactinoma, even when dopaminergic medication fails, a spectacularly spontaneous pregnancy can occur. This fact is the result of an “imperfect” suppression of the GnRH-FSH/LH-ovarian axis induced by permanent high PRL, which makes it possible for ovulation “escape”. 

The reason why hyperprolactinemia caused infertility was hypogonadotropic hypogonadism (HH), probably via blocking KNDy neurons. However, Oride et al. [44] demonstrated the sporadic restart of menstrual cycles in 25% of women with HH. In addition, in some cases of HH, the serum levels of gonadotropin increased sporadically during follow-up, regardless of the recovery of menstruation. This implies that it is possible to conceive spontaneously even in women with HH.

During normal gestation, the maternal PRL blood concentration rises from a basal level of 100–250 μIU/mL (beginning around 8 weeks) to 2000–4000 μIU/mL at term [45]. Responsible for its secretion is the maternal pituitary gland, where the PRL-secreting lactotrophs increase up to 50% of the cell population at term [46], and, to a smaller degree, the decidua [46,47]. PRL levels decline rapidly following delivery, reaching baseline within 1–3 weeks postpartum in non-lactating women [46]. The progressive increment in estrogen levels during pregnancy reduces dopamine’s (DA) release from hypothalamic tuberoinfundibular dopaminergic neurons (TIDA) and thus generates a continuous increase in PRL levels [5,48]. Furthermore, estrogen has a hyperplasic effect on pituitary lactotrophs [49] and directly stimulates PRL gene expression in these cells, with both actions increasing hormone secretion [26]. In microprolactinomas with high PRL, as in our case, the hormone levels only increase slightly during pregnancy because a very powerful lactophore activity already exists, with the pregnancy-induced endocrine modifications having a reduced (if any) impact on serum PRL [50].

The decidua produces the existing PRL in the amniotic fluid [49]; the peptide plays fundamental roles in local fluid and electrolyte regulation as well as the modulation of pro- and anti-inflammatory cytokines [51,52]. Prolactin levels in the amniotic fluid are extremely high, reaching up to 120 mIU/mL at the end of the second trimester, followed by a decrease to 8–16 mIU until term [46]. The decidual secretion of prolactin is insensitive to dopamine agonist administration because, despite the sudden decrease in maternal blood levels of PRL induced by DA, the hormone’s concentration in the amniotic fluid remains unchanged [45]. Therefore, the dynamic characteristics of local fluid and electrolytes are maintained, ensuring normal fetal growth and development [45]. 

With the appropriate treatment, only a small percentage, 7.7% [52], of microprolactinomas grow during pregnancy, and even fewer, 2–3%, become symptomatic and need supplementary therapy [7]. Furthermore, although almost no teratogenic action has been observed in response to bromocriptine and cabergoline [52,53,54,55], the standard therapy approach is to use DA for allowing ovulation and, if pregnancy occurs, to stop administration and carefully monitor tumor growth [38]. This, along with the treatment guidelines of the European Society of Endocrinology [56], was the reason for our decision not to administer DA during pregnancy and simply survey the tumor evolution with or without the occurrence of symptoms. No tumor-induced symptoms and no modifications of either PRL levels or MRI were recorded during pregnancy. 

However, if symptoms occur, and in cases of invasive or extracellular prolactinomas, the decision to continue DA must be individualized [53,57,58]. Another aspect that clinicians must be aware of is the possibility of an incidental occurrence of symptomatic pituitary apoplexy in all trimesters of pregnancy, a situation that requires prompt radiological, endocrinological and ophthalmological assessment and treatment [59].

The current medical literature reveals that hyperprolactinemia due to non-tumoral causes is completely remitted after delivery, while hyperprolactinemia due to microadenomas is remitted in up to 70% of cases [53]. The rate of resolution for spontaneous prolactinomas after pregnancy is about 27–29% [60,61,62]. This was not the case for us: the volume of pituitary gland tumors at 6 months was not significantly modified, and serum PRL remained at a high value.

To our knowledge, the few published cases of patients with hyperprolactinemia who developed spontaneous pregnancies were either microprolactinomas with mild to moderate elevations of serum prolactin (up to 100 ng/mL or 2127 μIU/mL) [39,63] or macroprolactinomas in which apoplexy occurred prior to pregnancy or during pregnancy, spontaneously normalizing or reducing prolactin levels [64], or with significantly lower levels of serum prolactin than our case [65]. 

As mentioned in protocols described in the literature, we considered that breastfeeding was safe, and we arrested it only at the patient’s request 6 months after delivery. PRL and MRI revealed no significant modifications during nursing.

## 4. Conclusions

Even when DA fails in amenorrheic women over a long period secondary to a microprolactinoma with high PRL plasma levels, the GnRH-FSH/LH-ovarian axis can be “imperfect”, and completely unexpected gestation can occur. 

There is not always a relationship between the tumor’s volume and its prolactin release. Furthermore, it can significantly vary in its endocrine activity without modifying its dimensions.

Guidelines for the treatment of small, asymptomatic prolactinoma during pregnancy do not recommend DA treatment. Furthermore, breastfeeding is allowed if a patient wants to do so.

The hypogonadotropic hypogonadism induced by high PRL is mainly manifested by low LH, and, as in this situation, the normal level of FSH and estradiol does not always induce follicle recruitment and development without abnormalities on the ovarian ultrasound.

## Data Availability

The data presented in this study are available on request from the corresponding author.
